# Face masks and fake masks: the effect of real and superimposed masks on face matching with super-recognisers, typical observers, and algorithms

**DOI:** 10.1186/s41235-024-00532-2

**Published:** 2024-02-02

**Authors:** Kay L. Ritchie, Daniel J. Carragher, Josh P. Davis, Katie Read, Ryan E. Jenkins, Eilidh Noyes, Katie L. H. Gray, Peter J. B. Hancock

**Affiliations:** 1 School of Psychology, University of Lincoln, Brayford Pool, Lincoln, LN6 7TS UK; 2 Psychology, Faculty of Natural Sciences, University of Stirling, Stirling, UK; 3 School of Psychology, Faculty of Health and Medical Sciences, University of Adelaide, Adelaide, Australia; 4 School of Human Sciences, Institute of Lifecourse Development, University of Greenwich, London, UK; 5 School of Human and Health Sciences, University of Huddersfield, Huddersfield, UK; 6 School of Psychology and Clinical Language Sciences, University of Reading, Reading, UK

**Keywords:** Face masks, Face matching, Super-recognisers, Automatic face recognition

## Abstract

Mask wearing has been required in various settings since the outbreak of COVID-19, and research has shown that identity judgements are difficult for faces wearing masks. To date, however, the majority of experiments on face identification with masked faces tested humans and computer algorithms using images with superimposed masks rather than images of people wearing real face coverings. In three experiments we test humans (control participants and super-recognisers) and algorithms with images showing different types of face coverings. In all experiments we tested matching concealed or unconcealed faces to an unconcealed reference image, and we found a consistent decrease in face matching accuracy with masked compared to unconcealed faces. In Experiment 1, typical human observers were most accurate at face matching with unconcealed images, and poorer for three different types of superimposed mask conditions. In Experiment 2, we tested both typical observers and super-recognisers with superimposed and real face masks, and found that performance was poorer for real compared to superimposed masks. The same pattern was observed in Experiment 3 with algorithms. Our results highlight the importance of testing both humans and algorithms with real face masks, as using only superimposed masks may underestimate their detrimental effect on face identification.

**Supplementary Information:**

The online version contains supplementary material available at 10.1186/s41235-024-00532-2.

## Introduction

### Unfamiliar face matching

While humans are very good at recognising the faces of familiar people (e.g. Bruce, 1986; Bruce et al., 2001; Burton et al., 1999), we are far poorer at recognising unfamiliar people. In a typical face matching task, participants are shown two images and are asked to judge whether they depict the same person or two different people. Unfamiliar face matching performance has been shown to be poor both in the laboratory (Clutterbuck & Johnston, 2002, 2004; Megreya & Burton, 2008; Ritchie et al., 2015, 2021, 2023; Sandford & Ritchie, 2021), and in live tasks matching a physically present unfamiliar person to a photograph (Davis & Valentine, 2009; Kemp et al., 1997; Megreya & Burton, 2008; Ritchie et al., 2020). Unfamiliar face matching performance is poor even in people who are employed to make identity decisions from images, such as checkout assistants (Kemp et al., 1997), passport officers (White et al., 2014), and police officers (Burton et al., 1999).

The addition of everyday paraphernalia such as glasses and sunglasses to one image in the pair has been shown to reduce face matching accuracy (Graham & Ritchie, 2019; Kramer & Ritchie, 2016; Noyes et al., 2021). Face masks have also been shown to impair face identification (Fitousi et al., 2021; Freud et al., 2020, 2021) and face matching (Carragher & Hancock, 2020; Dhamecha et al., 2014; Estudillo et al., 2021; Noyes et al., 2021), with masks causing more of a reduction in accuracy than sunglasses (Noyes et al., 2021). It is not clear, however, precisely why face masks cause an impairment to face matching performance. The current study seeks to shed light on the mechanisms underlying this effect by testing face matching using different types of lower face occlusions.

### Super-recognisers

Although unfamiliar face matching is generally poor, some people are able to perform with far higher accuracy than the general population. First described as having exceptional face memory (Russell et al., 2009), these people are referred to as super-recognisers (see Noyes et al., 2017 for a review). Although there are individual differences between super-recognisers, at the group level they perform with consistently higher accuracy than control participants (Bobak et al., 2016a, 2016b; Bobak et al., 2016a, 2016b; Davis et al, 2019; Noyes et al., 2018; Phillips et al., 2018). A recent study showed that super-recognisers are also more accurate than control participants at face matching with images wearing face masks (Noyes et al., 2021). The current study extends this work by testing both control participants and super-recognisers with different types of face coverings.

### Algorithms

In recent years, there has been a rapid improvement in the performance of facial recognition algorithms through the use of ‘Deep Convolutional Neural Networks’ (DCNNs; e.g. Cao et al., 2018; Kemelmacher-Shlizerman et al., 2016; Taigman et al., 2014). One study tested algorithms made in 2015, 2016 and 2017 and showed a monotonic increase in performance from the oldest (68% accurate) to the newest (96% accurate; Phillips et al., 2018). Face masks present a new challenge for algorithm face identification. A recent competition receiving 18 submissions found that eight did not meet the baseline criterion for verification errors (Boutros et al., 2021). The National Institute of Standards and Technology (NIST) in the USA runs a regular Face Recognition Vendor Test (FRVT) which is a standard test of facial recognition algorithms. The FRVT has consistently reported improvements in algorithm face identification with algorithms achieving higher accuracy than humans (NIST, 2022a). NIST now also runs an ‘FRVT Face Mask Effects’ looking specifically at algorithm identification from masked faces. Algorithms are presented faces with superimposed masks and are tasked with identifying the person from a database of unmasked images (NIST, 2022b). Updates to the test show that some developers have adapted their algorithms to better cope with face masks, although the shape, colour, and coverage of the different masks used in the test affects some algorithms’ ability both to detect the face in the first place, and then to correctly identify the person pictured (Ngan et al., 2022).

### Types of face coverings

While some previous studies of human face identification ability with face masks have used images of people wearing real masks (Dhamecha et al., 2014; Fitousi et al., 2021; Noyes et al., 2021), the majority have used pre-existing images with masks superimposed on to them (Carragher & Hancock, 2020; Estudillo et al., 2021; Freud et al., 2020, 2021). Some recent computer vision research has used real face masks (e.g. Jeevan et al., 2022; Lionnie et al. 2021), but the NIST FRVT Face Mask Effects test uses superimposed masks as the test images (Ngan et al., 2022).

It is not clear whether superimposed and real face masks produce different deficits in either human or computer face matching performance, and this difference is important for both theoretical understanding of face perception, and for understanding the impact of masks in applied face recognition practice. We have previously argued that one study using real face masks (Noyes et al., 2021) found a smaller reduction in face matching accuracy than a study using superimposed face masks (Carragher & Hancock, 2020) because it is possible that some elements of the person’s real face shape are still available to the viewer in real mask images but are covered in superimposed mask images. Although we predominantly use face texture to recognise other people (e.g. Burton et al., 2005), some element of face shape information may be useful (Rogers et al., 2022). Alternatively, it is possible that real face masks introduce extra texture information which may be more disruptive for face processing than superimposed masks, and the previously observed differences in findings (Carragher & Hancock, 2020; Noyes et al., 2021) were simply due to different task demands and methodologies.

### The current studies

It is not clear exactly why face masks cause such a marked impairment in human face matching performance. One possibility is that masks cover facial features that are useful for identification (Towler et al., 2017). But previous research suggests that the upper half of the face, which remains visible when wearing a face covering, tends to be more useful for identification than the lower half (Fisher & Cox, 1975; McKelvie, 1976). Alternatively, covering the features of the lower face might interfere with the holistic processes that are used in face recognition (Maurer et al., 2002; Tanaka & Farah, 1993). In support of this possibility, Freud et al. (2020) report that holistic processing is impaired for faces wearing a face mask (see also Stajduhar et al., 2021). However, face matching can be aided by featural comparisons (Towler et al., 2017; White et al., 2015), which can occur without holistic processing (Towler et al., 2021). Recent research has shown that featural comparisons can lead to modest improvements in masked face matching performance (Carragher et al., 2022). The final possibility considered here is that the face mask serves as a source of distraction by attracting attention to the mask and away from the visible facial features.

In Experiment 1, we compare human unfamiliar face matching with different types of superimposed lower face occlusions. In Experiment 2, we compare unfamiliar face matching by control participants and super-recognisers with superimposed and real face masks, and in Experiment 3, we test algorithm performance with the real and superimposed masks.

#### *Experiment 1*: *face matching with different types of superimposed lower face occlusions*

This experiment was designed to investigate whether different types of superimposed face masks modulate the degree of impairment caused to unfamiliar face matching performance. In a within-participants design, observers completed a matching task in which one face in each pair was always presented unmasked, while the other face was selected from the following mask conditions: control (unmasked), fitted mask (the mask closely followed the shape of the face), loose mask (the mask occluded a large square shape, including the neck) and the top half only (the entire lower half of the image was removed). First, we expect that performance will be higher for the control condition than all others, replicating the basic finding that face masks impair matching performance (Carragher & Hancock, 2020; Noyes et al., 2021). Comparisons between the mask conditions could potentially reveal the mechanism by which masks impair face matching performance. Higher accuracy in the fitted mask condition compared to the loose mask condition would suggest that observers can extract information about facial shape from the mask. Alternatively, significantly better performance in the top half only condition compared to the two mask conditions (fitted, loose), would suggest that masks are a source of attentional distraction. Finally, no difference between the three manipulated conditions (fitted mask, loose mask, top only) would be consistent with two different explanations; either that face masks impair matching performance because they cover facial features that are important for identification, or because they impede holistic processing. These final possibilities are inextricably linked because covering facial features will, by definition, also interfere with holistic processing.

## Method

### Participants

From a convenience sample of volunteers recruited via email and social media, we received complete data from 79 participants (22 male, 57 female; mean age: 34 years; SD: 16 years; range: 18–67 years). All participants were naïve to the aims of the study. This research was approved by the General University Ethics Panel at the University of Stirling, and all participants gave informed consent.

### Stimuli

**Fig. 1:**
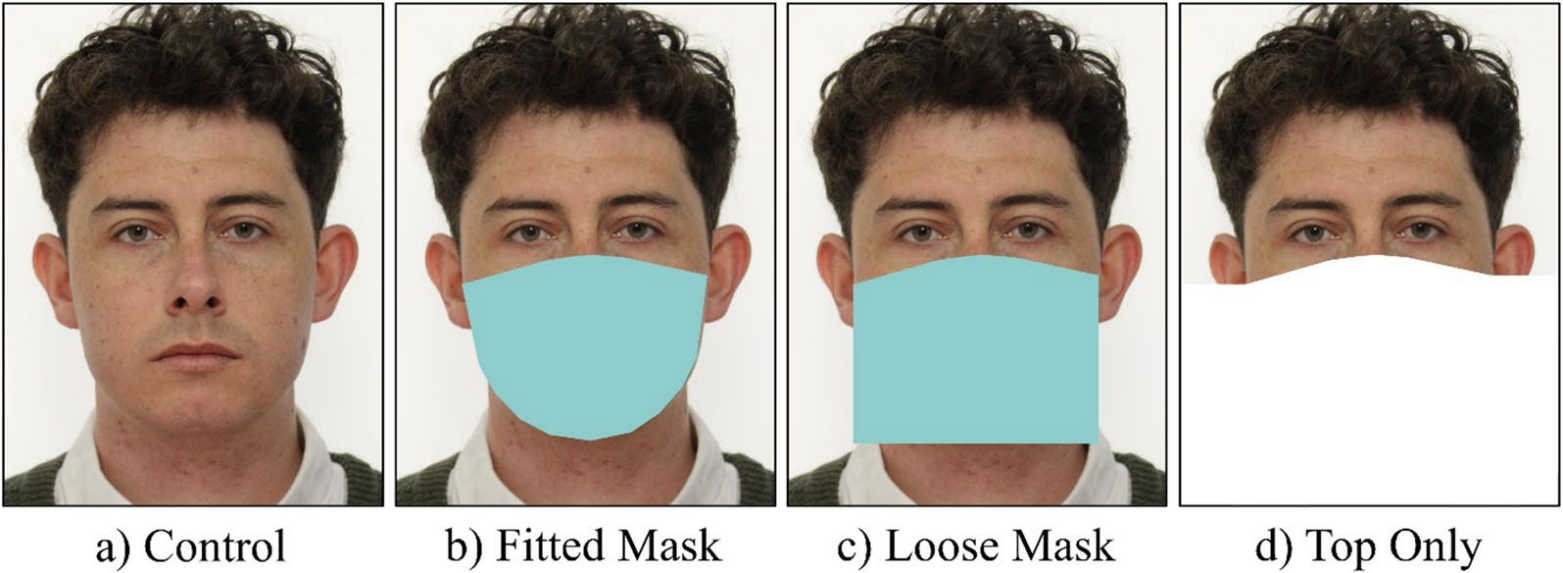
Examples of the **a** Control **b** Fitted Mask **c** Loose Mask and **d** Top Only stimuli used in Experiment 1. The images depict an identity who was not included in the experiment, but has given permission for their images to be used

The face masks in the current study were plain colour patches that were fitted to the faces automatically using custom written code (see Fig. 1). Automatically located landmark points were fine-tuned manually. The same landmark points below the eyes and over the bridge of the nose were used to establish the top of the mask in each mask condition (fitted, loose, top only). The fitted mask was created by filling the landmark points that follow the shape of the jaw with a plain pale blue patch (RGB 143, 205, 205), which is most similar to the FRVT Face Mask Effects’ ‘wide, medium coverage’ mask (Ngan et al., 2022). The loose masks were created by extending the occlusion 10 pixels down below the bottom of the jaw, square below the widest point at the ears. The top only condition was created by cropping the image below the top of the mask.

The faces for the current experiment came from two separate face matching tests. Half of the trials were the unfamiliar face pairs from the Stirling Famous Face Matching Task created by Carragher and Hancock (2020), making this the Stirling Unfamiliar Face Matching Task (SUFMT). These face pairs are images of amateur models that were downloaded from various online sources. The SUFMT consists of 40 image pairs, of which 20 are identity matches. The match and mismatch trials are evenly split for face sex. Each image only appears once within the SUFMT. The remaining trials came from the short version of the Kent Face Matching Test (KFMT; Fysh & Bindemann, 2018). The KFMT also consists of 40 trials, of which 20 are matches and 20 are mismatches. Each image pair consists of one smaller image that is typical of a student ID card, and one larger high-quality portrait image. The KFMT also consists of male and female face pairs. Thus, the experiment consisted of 80 trials in total, of which 40 were identity matches.

Trials from the SUFMT and KFMT were intermixed and randomised. Because all participants completed the same two tasks, we did not compare performance between the two tests. Allocation of trial pairs to mask conditions (control, fitted mask, loose mask, top only) was randomised between participants, such that all pairs were presented in each mask condition across participants. All participants completed 20 trials of each mask condition, of which 10 were match trials and 10 were mismatch trials. Face pairs in the fitted mask, loose mask and top only conditions consisted of one full-view face and one altered face. This image arrangement is consistent with the scenario in which a masked individual presents an official photo-ID document for inspection. In the KFMT, the smaller ID image was always unmasked, while the larger image was shown in each mask condition. All images were presented in colour. Images from the SUFMT were 420 × 595 px in size. Images from the KFMT were presented in their original sizes (Fysh & Bindemann, 2018); small (142 × 192 px), large (283 × 332 px).

### Procedure

Participants completed the experiment on their personal computers via a web link. The experiment was run using Qualtrics survey software. Participants were informed that their task was to decide whether the two simultaneously presented images showed the same person or two different people. Responses were made using a 6-choice scale, which conveyed the identification decision (“Same”, “Different”) and confidence (“Certain”, “Think”, “Guess”). There was no time limit to give a response. All trial types were intermixed and presented in a random order in a single experimental block that consisted of all 80 trials. The experiment took approximately 15 min (*M* = 899 s, SD = 363 s) to complete.

### Analysis

We analysed the data using signal detection measures of sensitivity (d′) and response bias (criterion). Sensitivity measures how well participants can discriminate match pairs from mismatches, with higher values indicating better performance (Macmillan & Creelman, 2004). Criterion is a measure of response bias, which shows whether participants had an overall tendency to report that pairs were a match (“same”) or mismatch (“different”). Positive criterion values indicate a bias to respond “different” across all trials (i.e. a conservative criterion), whereas negative values signal a “match” response bias (i.e. a liberal criterion). To calculate both measures, we collapsed across the confidence component of our scale, leaving only “same” and “different” responses (e.g. “Certainly Same”, “Think Same” and “Guess Same” were counted as “same”). These simplified responses correspond to hits (correctly responding “same” on a match trial) and false alarms (incorrectly responding “same” on a mismatch trial) which are used to calculate both d′ and criterion (Macmillan & Creelman, 2004; Stanislaw & Todorov, 1999). In both Experiment 1 and 2, we corrected for hits of 1 using the formula 1–1/(2N) and false alarms of 0 using the formula 1/(2N) where N is the number of trials in each condition. The number of trials was the same in each condition in each experiment, giving a maximum d′ value of 3.29. In addition to traditional frequentist hypothesis testing, we included Bayes factors calculated in JASP (JASP Team, 2020) with default prior width, which allowed us to quantify the extent to which the data support the alternative hypothesis (BF_10_). We interpret BFs of less than 3.0 as anecdotal evidence of the alternative hypothesis (e.g. Jeffreys, 1961).

## Results and discussion

All data for all experiments is available at https://osf.io/qgxhs/?view_only=6c6e8368c49d4d4fb634ada0671a7972

We present descriptive statistics here for ease of reading—full analysis of accuracy as defined by per cent correct can be found in the Additional file 1. In Experiment 1, face matching accuracy in each condition varied as follows: control (no concealment), 40% to 95% out of 20 (*M* = 69%, SD = 11%); fitted mask, 35% to 85% (*M* = 62%, SD = 10%); loose mask, 30% to 85% (*M* = 61%, SD = 12%); and top only, 30% to 90% (*M* = 60%, SD = 12%).

### Sensitivity

**Fig. 2:**
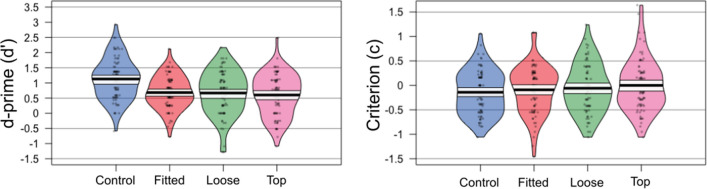
Sensitivity (*d*′) and criterion scores for Experiment 1

Our main analysis uses signal detection theory as is common in the literature. A repeated measures ANOVA revealed a significant effect of mask condition on d′, *F*(3, 234) = 13.55, *p* < 0.001, *η*_*p*_^2^ = 0.15, BF_10_ > 1000 (see Fig. 2). Bonferroni corrected post-hoc comparisons showed that sensitivity was significantly higher in the control condition compared to all other conditions (all *p*s < 0.001, all BF_10_ > 400), which did not differ from each other (all *p*s > 0.999, all BF_10_ < 1). The pattern of results is the same when the results are analysed using per cent correct, for both overall accuracy (collapsing across match and mismatch trials), and for match trials. However, there was no effect of mask condition on mismatch trials accuracy (see Additional file 1: Sect. 1).

### Criterion

There was a non-significant effect of mask condition on response bias, *F*(3, 234) = 2.12, *p* = 0.098, *η*_*p*_^2^ = 0.03, BF_10_ = 0.22.

Sensitivity was highest in the control condition and fell significantly for the three mask conditions, which did not differ from each other. These results suggest that the shape of the superimposed mask does not influence the degree of impairment to matching performance. Our findings suggest that masks impair performance either because they occlude facial features that carry identity information, or because they disrupt holistic processing. However, this experiment only examined the effect of superimposed masks. It is possible that real masks introduce extra information, either attracting attention to the mask, or adding additional spurious texture information to the face. Therefore, it is possible that images of faces wearing real face masks may lead to reduced face matching ability compared to superimposed masks. Alternatively, as we have previously suggested (Noyes et al., 2021), it is possible that real face masks might preserve some information about face shape, which could be useful for identification (see Rogers et al., 2022). Therefore, in the following experiment we tested unfamiliar face matching with real and superimposed face masks.

#### *Experiment 2*: *face matching with real and superimposed masks*

This experiment tested both typical participants and super-recognisers. Both sets of participants were recruited from a large database of participants used in previous research (e.g. Belanova et al., 2021; Noyes et al., 2021; Satchell et al., 2019). Importantly, none of the participants who took part in this study had taken part in our previous test of masked face matching (Noyes et al., 2021). Here we aimed to examine the effect of real and superimposed masks on typical participants’ and super-recognisers’ unfamiliar face matching performance.

Previous research using super-recognisers has tended to assess their ability using two tests: the Glasgow Face Matching Test: short version (GFMT, Burton et al., 2010) and the Cambridge Face Memory Test: Extended (CFMT + , Russell et al., 2009). The GFMT has recently been criticised for being a relatively easy test (e.g. Ramon, 2021), therefore here, we add a third test to the initial recruitment battery, the Kent Face Matching Test (KFMT, Fysh & Bindemann, 2018), which is a more difficult test of face matching than the GFMT.

Our super-recognisers are defined as individuals scoring 100% (40 out of 40) on the GFMT (Burton et al., 2010), 93% (95 or more out of 102) on the CFMT + (Russell et al., 2009) and 82.5% (33 or more out of 40) on the KFMT (Fysh & Bindemann, 2018). Less than 5% of people achieve perfect performance on the GFMT (Burton et al., 2010), while an estimated 2% score 95 or above on the CFMT + (Bobak et al., 2016a, 2016b; Russell et al., 2009), and average performance on the KFMT is 66.22%, taking the mean of performance reported in three studies (Fysh, 2018; Fysh & Bindemann, 2018; Gentry & Bindemann, 2019).

During the original database recruitment process, many participants did not meet the criteria to be classed as super-recognisers. Typical-ability participants were invited from this second group who had previously scored within approximately 1 standard deviation of the normal population mean on the GFMT (i.e. 28–36: Burton et al., 2010), CFMT + (i.e. 58–83: Bobak et al., 2016a, 2016b) and the KFMT (i.e. 24–29: Fysh, 2018; Fysh & Bindemann, 2018; Gentry & Bindemann, 2019).

## Method

### Participants

The control group were recruited from a large database of interested participants from the UK used in previous research (Belanova et al., 2021; Noyes et al., 2021; Satchell et al., 2019). We received complete data from 175 control participants (55 male, 118 female, 2 other; mean age 45 years; SD: 14 years; age range 18–75 years). The control participants had a mean GFMT score of 33.89/40 (SD = 2.06), a mean CFMT + score of 73.17 (SD = 6.81), and a mean KFMT score of 27.05 (SD = 1.60) as assessed in a previous battery of unpublished tests.

The super-recognisers were recruited from the same large database as the control participants. We received complete data from 136 super-recognisers (43 male, 91 female, 2 other; mean age 39 years; SD: 9 years; age range 24–60 years). The super-recognisers all scored 40/40 on the GFMT, had a mean CFMT + score of 97.32 (SD = 1.97), and a mean KFMT score of 34.90 (SD = 1.53) as assessed in a previous battery of unpublished tests. No participants were given monetary compensation for taking part. The experiment received ethical approval from the University of Reading (ref: 2021–093-KG).

### Stimuli

**Fig. 3:**
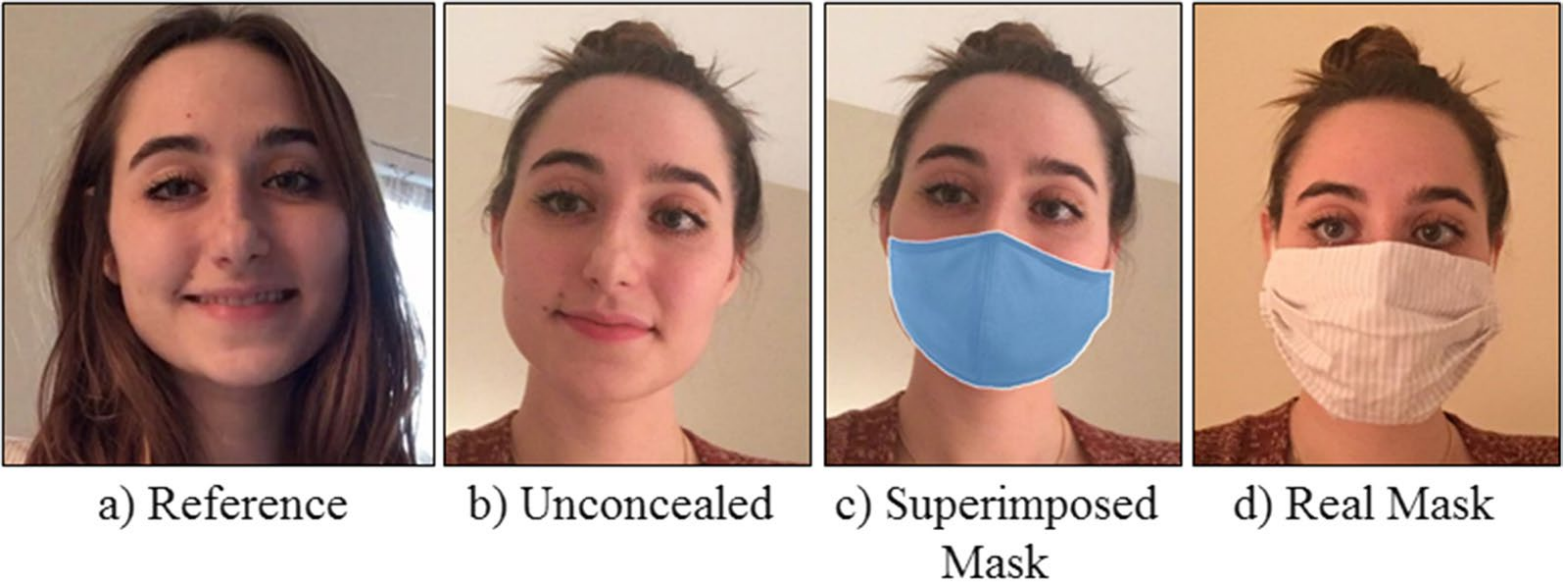
Examples of the **a** Reference **b** Unconcealed **c** Superimposed Mask and **d** Real Mask stimuli used in Experiment 2. The images depict an identity who was not included in the experiment, but has given permission for their images to be used

The stimuli were images of people who had volunteered photographs of themselves for this research project. Models were recruited from the same large database as the participants, and none of the models also acted as participants. Models were asked to provide multiple images of themselves both with and without face masks. The images supplied by 60 models (21 male, 39 female) were used to create the stimuli pairs in four concealment conditions: a) reference image (unconcealed), b) unconcealed image, c) superimposed mask image (this was the unconcealed image (b) with a face mask superimposed on to the face), and d) real mask image (see Fig. 3). Reference images always depicted the identity with a different background to the unconcealed and real mask images. We did not remove the backgrounds from the images, therefore the same background in the reference and test images may have provided a cue that the images showed the same person. As in our previous research on face matching with masked faces (Noyes et al., 2021), the unconcealed reference image chosen for each model was front-facing and showed a neutral expression (where possible). A different identity ‘foil’ image was selected from the same model database for each identity to serve as the reference image in mismatch trials. The foil identities were chosen to match the same verbal description as the target identity e.g. “young woman, dark hair”. A subset of 20 of the identities was used in a recent study of forensic facial examiners (Noyes et al., 2024).

Superimposed masks were added to the unconcealed images by open source software (Anwar & Raychowdhury, 2020https://github.com/aqeelanwar/MaskTheFace) that uses standard face landmarking code to locate the relevant part of the face and superimpose a mask image. A variety of mask types are available; we used the standard surgical mask, as illustrated in Fig. 3c. This mask is most like the NIST FRVT Face Mask Effects ‘wide, medium coverage’ mask which is particularly important for Experiment 3 which uses these same stimuli.

### Procedure

The stimuli were presented side by side in pairs. In all trials, the image on the left was the reference image for match trials, and the foil image for mismatch trials. The image on the right was either the unconcealed, superimposed mask, or real mask image. The assignment of identities to conditions was counterbalanced between participants, and each participant saw each identity only once. Participants saw ten trials in each concealment condition (unconcealed, superimposed mask, real mask) for each trial type (match, mismatch), making a total of 60 trials. On each trial, participants were asked to indicate whether the two images showed the same person or two different people.

## Results and discussion

Again, we present descriptive statistics here for ease of reading—full analysis of accuracy as defined by per cent correct can be found in the Additional file 1. In Experiment 2, as a group, the face matching scores (out of 60) for super-recognisers (range = 44–60, *M* = 54, SD = 3) were higher than controls (range = 37–57, *M* = 48, SD = 4). Accuracy across both groups of participants in each condition was as follows: unconcealed, (range = 55–100%, *M* = 89%, SD = 10%); superimposed mask, (range = 50–100%, *M* = 83%, SD = 10%); and real mask, (range = 45–100%, *M* = 81%, SD = 11%).

### Sensitivity

**Fig. 4:**
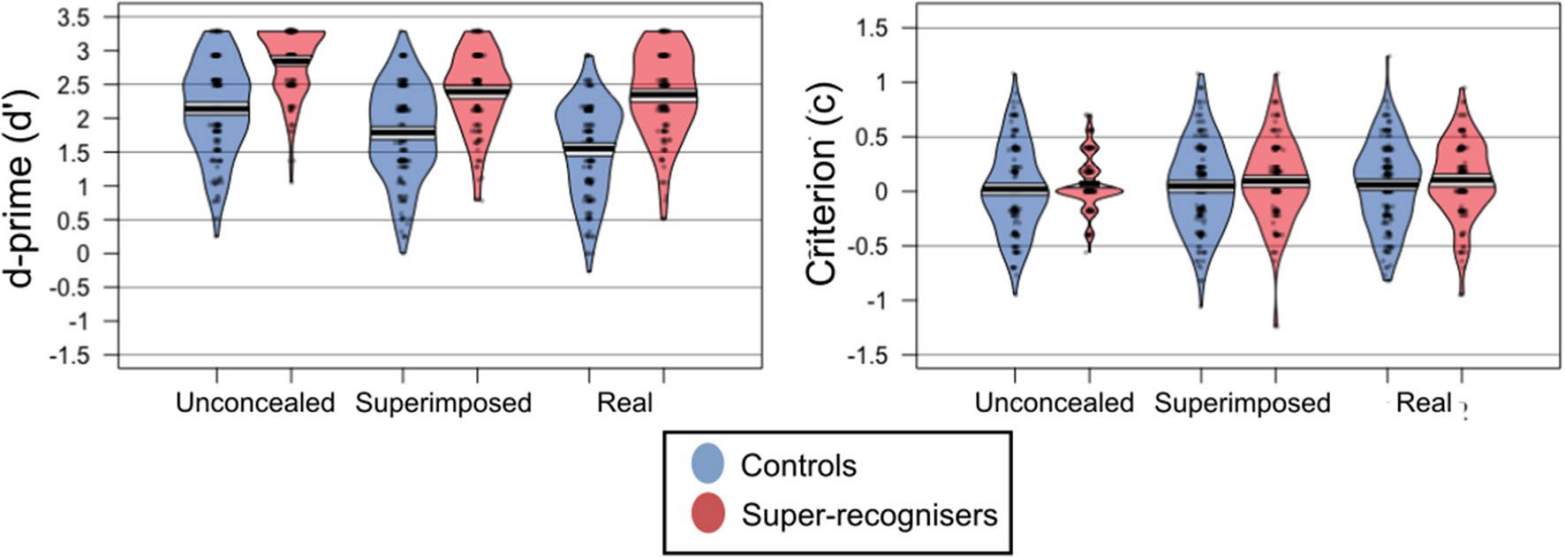
Sensitivity (*d*′) and criterion scores for Experiment 2

As in Experiment 1 our main analysis uses signal detection theory. Again, we corrected for hits of 1 and false alarms of 0, giving a maximum d′ value of 3.29. A mixed ANOVA with the within subjects factor of mask condition (unconcealed, superimposed mask, real mask) and the between subjects factor of participant group (control, super-recogniser) revealed a significant effect of mask condition on d′, *F*(3, 618) = 66.06, *p* < 0.001, *η*_*p*_^2^ = 0.18, BF_10_ > 1000, see Fig. 4. Bonferroni corrected post-hoc comparisons showed that sensitivity was significantly higher in the unconcealed condition (*M* = 2.45, SD = 0.70) compared to both the superimposed mask condition (*M* = 2.05, SD = 0.71, *t*(310) = 8.77, *p* < 0.001, BF_10_ > 1000), and the real mask condition (*M* = 1.90, SD = 0.78, *t*(310) = 10.68, *p* < 0.001, BF_10_ > 1000). The comparison between superimposed and real masks was also significant, whereby sensitivity was higher with superimposed compared to real masks, *t*(310) = 3.08, *p* = 0.006, BF_10_ = 6.51. There was a significant main effect of participant group whereby the super-recognisers as a group performed more accurately (*M* = 2.53) than the control participants (*M* = 1.83), *F*(3, 309) = 224.36 *p* < 0.001, *η*_*p*_^2^ = 0.42, BF_10_ > 1000. The interaction was non-significant *F*(3, 618) = 1.98, *p* = 0.139, *η*_*p*_^2^ < 0.01, BF_10_ = 0.59).

The pattern of results is the same when the results are analysed using per cent correct, for overall accuracy (collapsing across match and mismatch trials), match, and mismatch trials (see Additional file 1: Sect. 2).

### Criterion

A mixed ANOVA with the within subjects factor of mask condition (unconcealed, superimposed mask, real mask) and the between subjects factor of participant group (control, super-recogniser) showed a non-significant main effect of mask condition on response bias, *F*(3, 618) = 2.12, *p* = 0.225, *η*_*p*_^2^ < 0.01, BF_10_ = 0.04. There was a non-significant main effect of participant group, *F*(3, 309) = 1.96, *p* = 0.163, *η*_*p*_^2^ < 0.01, BF_10_ = 0.23 and a non-significant interaction *F*(3, 618) = 0.01, *p* = 0.994, *η*_*p*_^2^ < 0.01, BF_10_ < 0.01.

In this experiment we found that human observers performed most accurately with unconcealed faces, then with superimposed masks, and were least accurate with real face masks. These results demonstrate the importance of the face covering used when testing face matching ability. Both superimposed and real face masks impaired performance, possibly because they attract attention to the mask, or because they disrupt holistic processing. It is unclear why real face masks impaired performance more than superimposed masks, but this could be a result of spurious texture information being introduced by the mask, disrupting face matching ability to a greater extent than superimposed masks. Experiment 3 tested the effects of both types of face masks on algorithm performance.

#### *Experiment 3*: *algorithm performance*

In this experiment, we tested four face recognition algorithms with face images covered by both superimposed and real face masks. We wished to repeat the pairings (reference image compared to unconcealed, real mask, and superimposed mask) shown to the human participants, for a direct comparison. However, as computers do not grow tired with time on task, we are able to test other pairings. In particular, we were interested in further exploring cases involving an unmasked reference image and a test image wearing a real mask. Carragher and Hancock (2020) reported that although Deep Convolutional Neural Networks (DCNNs) were able to accurately match faces with superimposed masks, their performance for pairs in which one face was unobstructed and the other was wearing a mask was far below that for pairs where both faces were masked. Therefore, might it help the algorithms to superimpose a fake mask on the reference image?

All four algorithms that we tested are DCNNs that make image computations in a broadly similar way. An input image of a face (here the images from our matching task) is processed to generate a vector of 512 real-value numbers that make up the system’s representation of that face image, sometimes termed an embedding. To decide whether two faces show the same identity, the two vectors are compared. If they are similar enough, the faces are declared a match. There is a variety of ways to compute the similarity of the two vectors: all the algorithms here use the cosine of the angle between the vectors. This gives a value of 1 for a perfect match, when the angle is zero, and zero when the vectors are orthogonal (90 degrees apart). The score can go negative, if the angle between the vectors is greater than 90 degrees.

The threshold for deciding that two faces match is a critical aspect of the system. A high threshold reduces the likelihood of declaring an incorrect match (i.e. a false positive). However, it also increases the likelihood of incorrectly rejecting a true match (i.e. a miss). An ideal algorithm would give complete separation between the similarity scores of match and mismatch pairs, with a threshold being set in the gap between the two distributions. In practice, when a DCNN is used with a large database there will likely be some overlap of these distributions, so a threshold is typically set to give an acceptable false positive rate, for example 1 in 10,000 comparisons. What is deemed acceptable will depend on the application and desired level of security. Here, we use the default recommended thresholds for each algorithm (as provided by the developers). Note that none of these algorithms had been designed specifically for use with masked faces. A mask only on one face seems likely to decrease the similarity score for a given pair (Carragher & Hancock, 2020), so there may be a case for using a lower threshold to declare a match in this circumstance, but we do not explore that possibility here.

## Method

### Stimuli

The stimuli were those used in Experiment 2. We also added an additional condition, in which we superimposed a fake mask onto the reference image and paired those with the real mask images. We therefore had four conditions: Unconcealed, Superimposed, Real, and Masked-Reference.

### Software

We tested four different automatic face recognition (AFR) algorithms, all based on deep convolutional networks. Two are (as yet) unpublished research algorithms, made available to us through the FACER2VM project (‘Face Matching for Automatic Identity Retrieval, Recognition, Verification and Management’ EPSRC grant no. EP/N007743/1); one from Imperial College London (ICL), the other from the University of Surrey (SU). The other two are FaceNet (Schroff et al., 2015) and ARCFace (Deng et al., 2019), as implemented in Deepface (https://github.com/serengil/deepface). These final algorithms were state of the art in their day and are included as an indication of how AFR performance is improving.

### Procedure

Each image was submitted to each AFR separately and the resultant vector stored. The four similarity scores (Reference–Unconcealed; Reference–Superimposed; Reference–Real; and Masked Reference–Real) were then computed locally in Matlab.

## Results

**Fig. 5:**
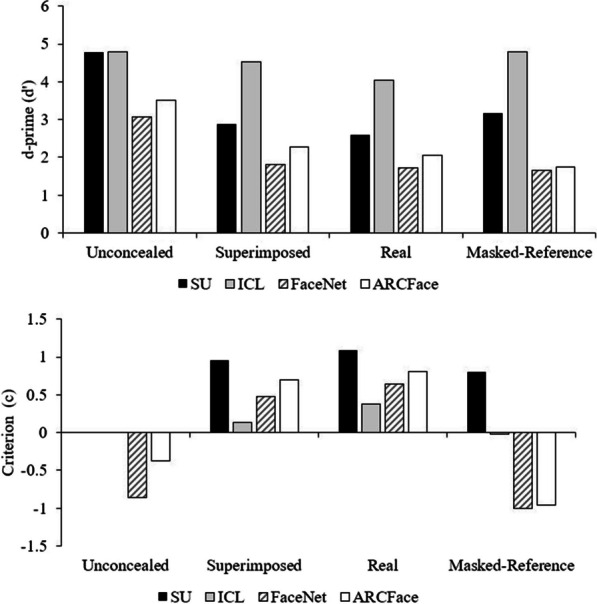
Sensitivity (*d*′) and criterion scores for the four AFR algorithms in the four test conditions. There are no error bars as the algorithms are deterministic

D-prime and Criterion scores are shown in Fig. 5. The two research algorithms achieve 100% accuracy in some conditions. This requires an adjustment to d′ that assumes half an error across the 60 trials, resulting in a maximum d′ of 4.79 (Stanislaw & Todorov, 1999).

### Sensitivity

There is a consistent order evident across the four algorithms, with the ICL system better than SU, which is better than the two older algorithms (and ARCFace is somewhat better than FaceNet). Note that inferential statistics cannot be conducted: the algorithms are deterministic so there is no variability to test. Adding a mask to the reference image improved sensitivity for the two research algorithms but not the older algorithms. Importantly for our research question, sensitivity for all four of the algorithms was lower for real face masks compared to superimposed masks.

### Criterion

The most obvious effect among the criterion values shown in Fig. 5 is that in the two masked conditions, the criterion for all the algorithms is increasingly conservative, meaning a shift towards reporting mismatch. Perhaps this result is not surprising, as none of these algorithms were designed to work with masks. With a mask across one face, the two faces appear more different to the AFR algorithms. Conversely, when a mask is added to the reference image, the criterion drops for all algorithms, going strongly negative for the two older algorithms. They see the mask on each face and interpret it as greater similarity between the two, resulting in a shift towards reporting a match, with little change in sensitivity. In the unconcealed condition, both research algorithms performed perfectly, resulting in zero bias. The two older algorithms have a negative criterion, indicating a bias towards reporting a match.

### Analysis of mask size

In Experiments 2 and 3 we have shown that both humans and algorithms are poorer at face matching with real masks compared to superimposed masks. We sought to determine whether, in our stimulus set, real masks covered a greater area of the face than the superimposed masks. We used sketchandcalc.com to determine the area of the face covered by the masks. A paired samples *t*-test showed that real masks (mean percentage of face covered = 48.17%) did cover more of the face than our superimposed masks (mean percentage of face covered = 39.38%) *t*(59) = 13.12, *p* < 0.001, Cohen’s d = 1.69, BF_10_ > 1000. To determine whether mask size explains performance, we correlated mask size with item accuracy (per cent of participants responding correctly to each item). For this analysis we used only control participant data as we did not find group differences in the main task. Mask area was not correlated with item accuracy *r*(118) = 0.02, *p* = 0.803, BF_10_ = 0.12. In addition we correlated change in mask size (real mask minus superimposed mask percentage of face covered) with change in accuracy per item (superimposed mask minus real mask accuracy) and found a non-significant correlation *r*(60) = − 0.01, *p* = 0.951, BF_10_ = 0.16. Mask size therefore does not explain accuracy on our task. Below we discuss possible explanations for our effects.

## Discussion

In three experiments, we have shown that face masks impair face matching performance for both typical human observers and super-recognisers, as well as four AFR algorithms, replicating previous work (Boutros et al., 2021; Carragher & Hancock, 2020; Dhamecha et al., 2014; Estudillo et al., 2021; Noyes et al., 2021). It is worth noting that we do not suggest that human observers and algorithms are equivalent or are performing the task in the same way. It is possible that humans approach this task in a way akin to a deep neural network, but this is a topic which requires further research. Importantly, irrespective of the mechanisms driving performance, Experiments 2 and 3 showed that both humans and algorithms are poorer at matching faces when one image in the pair wears a real face mask compared to a superimposed face mask. This highlights the importance of the type of face coverings used when testing both humans and computer algorithms. Our data suggest that the current tendency to rely on superimposed face coverings in research could be underestimating the degree of impairment real face masks cause in real-world settings.

In Experiment 1, sensitivity was highest in the control condition and fell significantly for the three mask conditions—which did not differ from each other. This finding suggests that the shape of the superimposed face covering does not influence the degree of impairment to matching performance. In Experiments 2 and 3, both humans and algorithms were more impaired in the real face mask condition than the superimposed mask condition. The explanation as to why face coverings impair face matching performance, and why real masks impair performance more than superimposed masks remains unclear. Real face masks are not standardised, and in this study each model identity wore their own face mask (we did not provide a standard mask). Superimposed masks, in contrast, are applied in a uniform way across faces. Real face masks therefore add more variability in a number of dimensions than superimposed masks. Each real face mask is fitted differently to each face, whereas the technique used here and elsewhere (Ngan et al., 2022) to fit superimposed masks to faces ensures a tight fit. In Experiment 1, a loose-fitting mask and even the complete removal of the bottom half of the image did not result in additional impairment beyond the fitted mask, and in Experiment 2 although we found that our real masks covered more of the face than the superimposed masks, mask size was not correlated with item accuracy. Therefore mask size alone does not explain our findings in Experiment 2 where real masks resulted in a larger impairment than superimposed masks. The variability of the fit of real masks is not captured with superimposed masks, which may introduce more noise, resulting in a greater impairment for real masks. Importantly, real masks introduce extra variability in terms of texture information to the face which may disrupt processing. It is also possible that in wearing a real face mask, other aspects of the face are slightly changed such as the ears are pulled forward, which may also produce greater variability in the images, resulting in the impairment in face matching which we see here. We would suggest that future research may wish to standardise real masks, for example by having every model wear a surgical mask of the same type. This would not overcome the issue of standard masks covering more of one person’s face than another, or more of the face than a superimposed mask, but would remove the variability in mask texture. These issues all highlight that real masks fit the face differently to superimposed masks, and emphasise the importance of using real face masks rather than superimposed masks for research and in applied settings.

In this paper, we sought to explore the different effects of real and superimposed masks on face matching performance. However, it is important to understand why either type of obstruction affects face matching performance. It is possible that both types of masks cover features that are useful for identification, interfere with holistic processing, and attract attention. The evidence for face masks attracting attention is mixed. One study found evidence from EEG that more attentional mechanisms are involved when viewing faces wearing masks compared to unconcealed faces (Żochowska et al., 2022). Another study, however, showed that gaze cueing is not affected by face masks (Dalmaso et al., 2021), suggesting that masks do not influence attention. In Experiment 1 here, the same impairment occurred when faces were masked (fitted or loose) as when the bottom half of the image was completely removed. These findings suggest that masks do not impair face matching performance because they attract attention. Instead, our findings suggest that masks impair performance either because they occlude facial features that carry identity information, or because they disrupt holistic processing (as in Freud et al., 2020; Stajduhar et al., 2021). We cannot separate these two possible explanations for our results because covering facial features necessarily also interferes with holistic processing. Further research is needed to disentangle these possibilities.

Crucial to our results is the finding that both humans and algorithms were poorer at face matching when the images showed people wearing real masks compared to superimposed masks. Comparing two of our previous studies, we found that one study using real face masks (Noyes et al., 2021) showed a smaller reduction in face matching accuracy than a study using superimposed face masks (Carragher & Hancock, 2020). We have not replicated this effect here, suggesting that differences between the results of the previous work may be due to different methodologies—Carragher and Hancock (2020) used a between subjects design with different participants in each mask condition, whereas participants in Noyes et al. (2021) participants all viewed each mask condition. The differences in results may also be due to variations in the baseline matching difficulty of the different identity sets used. This is evidenced when we look again at the original data. In the study using real masks (Noyes et al., 2021; Experiment 2), unconcealed unfamiliar face matching *d*-prime by controls = 1.10, dropping to 1.03 when one image wore a mask. The equivalent values for the study using superimposed masks (Carragher & Hancock, 2020) were *d*-prime = 2.74 for unconcealed faces and a substantially greater drop to 1.80 when one image had a superimposed mask. In the current study, we used the same identities in all conditions, overcoming the issue of different baseline difficulties in the tasks (Carragher & Hancock, 2020; Noyes et al., 2021).

Both super-recognisers and algorithms, in addition to control participants, were impaired at face matching by face coverings, particularly real face masks. This highlights the fact that face masks pose a problem for the very best humans as well as algorithms the likes of which are employed in security settings to perform face matching tasks. A recent study testing forensic face examiners (people who are employed to make face comparisons and whose evidence can be heard in court) showed that even with masked faces the examiners significantly outperformed controls on a face matching task (Noyes et al., 2024). Taken together these results demonstrate that there is a clear role for very high performing humans and algorithms in security settings, and although face masks reduce matching accuracy, algorithms and specialist humans outperform controls.

Our research further highlights the problem that face masks pose for identification, and also emphasises the importance of considering which types of face coverings are used when testing both humans and computers. Since real-world images will involve images of people wearing real face masks, our data suggest it is important to test humans and algorithms with real instead of superimposed masks, as a failure to do so may underestimate the problem posed by face masks.

### Supplementary Information

**Additional file 1**. Supplementary analyses.

## Data Availability

The datasets generated and/or analysed during the current study are available in the OFS repository https://osf.io/qgxhs/?view_only=6c6e8368c49d4d4fb634ada0671a7972
