# Supplementary material for: Face masks and fake masks: the effect of real and superimposed masks on face matching with super-recognisers, typical observers, and algorithms
Source: Cogn Res Princ Implic. 2024 Feb 2;9:5. doi: 10.1186/s41235-024-00532-2 (PMC10834892; doi:10.1186/s41235-024-00532-2)
Supplement: Supplementary file 1 — Additional file 1. Supplementary analyses. [file 41235_2024_532_MOESM1_ESM.docx]

Supplementary Materials

**Section 1. Experiment 1 – supplementary analyses**

In addition to the signal detection analyses presented in the main paper, we also carried out analyses of overall percent correct (collapsing across match and mismatch trials), and analyses of match and mismatch trials separately.

*Overall accuracy*

A repeated measures ANOVA on overall accuracy revealed a significant effect of mask condition on overall accuracy, *F*(3, 234) = 13.46, *p* < .001, *η_p_*^2^ = .15, BF_10_ > 1000 (see supplementary Figure 1a). Bonferroni corrected post-hoc comparisons showed that sensitivity was significantly higher in the control condition compared to all other conditions (all *p*s < .001, all BF_10_ > 500), which did not differ from each other (all *p*s > .999, all BF_10_ < 1).

**Supplementary Figure 1.** Supplementary data from Experiment 1 for a) overall percent correct (collapsing across match and mismatch trials; b) match trials; c) mismatch trials.


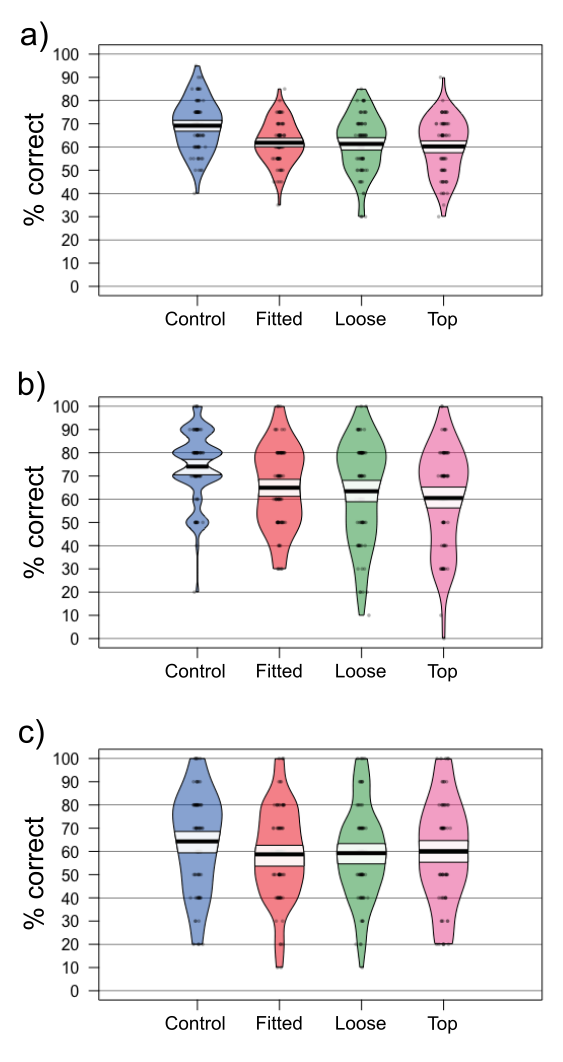


*Match trial accuracy*

A repeated measures ANOVA on match accuracy also revealed a significant effect of mask condition on match accuracy, *F*(3, 234) = 13.15, *p* < .001, *η_p_*^2^ = .14, BF_10_ > 1000 (see supplementary Figure 1b). Bonferroni corrected post-hoc comparisons showed that sensitivity was significantly higher in the control condition compared to all other conditions (all *p*s < .001, all BF_10_ > 200), which did not differ from each other (all *p*s > .150, all BF_10_ < 1).

*Mismatch trial accuracy*

A repeated measures ANOVA on mismatch accuracy showed a non-significant effect of mask condition, *F*(3, 234) = 1.80, *p* = .148, *η_p_*^2^ = .02, BF_10_ = 0.15 (see supplementary Figure 1c).

**Section 2. Experiment 2 – supplementary analyses**

Again, we carried out analyses of overall percent correct (collapsing across match and mismatch trials), and analyses of match and mismatch trials separately.

***Overall accuracy***

A mixed ANOVA with the within subjects factor of mask condition (unconcealed, superimposed mask, real mask) and the between subjects factor of participant group (control, super-recogniser) revealed a significant effect of mask condition on overall accuracy, *F*(3, 618) = 60.76, *p* < .001, *η_p_*^2^ = .16, BF_10_ > 1000, see Supplementary Figure 2a. Bonferroni corrected post-hoc comparisons showed that accuracy was significantly higher in the unconcealed condition (*M* = 88.55%) compared to both the superimposed mask condition (*M* = 83.05%, *t*(310) = 8.52, *p* < .001, BF_10_ > 1000), and the real mask condition (*M* = 80.88%, *t*(310) = 10.34, *p* < .001, BF_10_ > 1000). The comparison between superimposed and real masks was also significant *t*(310) = 2.96, *p* = .009, BF_10_ = 4.57. There was a significant main effect of participant group whereby the super-recognisers as a group performed more accurately (*M* = 89.73%) than the control participants (*M* = 79.83%, *F*(3, 309) = 207.03 *p* < .001, *η_p_*^2^ = .40, BF_10_ > 1000. The interaction was non-significant *F*(3, 618) = 1.94, *p* = .144, *η_p_*^2^ < .01, BF_10_ = 0.58).

**Supplementary Figure 2.** Supplementary data from Experiment 2 for a) overall percent correct (collapsing across match and mismatch trials; b) match trials; c) mismatch trials.


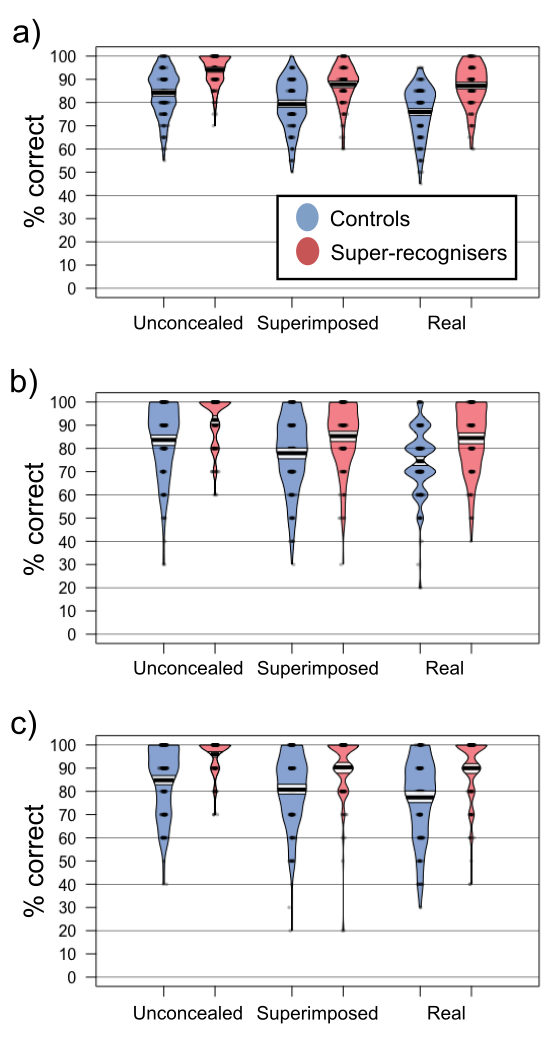


***Match trial accuracy***

A mixed ANOVA with the within subjects factor of mask condition (unconcealed, superimposed mask, real mask) and the between subjects factor of participant group (control, super-recogniser) revealed a significant effect of mask condition on match trial accuracy, *F*(3, 618) = 42.49, *p* < .001, *η_p_*^2^ = .12, BF_10_ > 1000, see Supplementary Figure 2b. Bonferroni corrected post-hoc comparisons showed that match accuracy was significantly higher in the unconcealed condition (*M* = 87.43%) compared to both the superimposed mask condition (*M* = 81.16%, *t*(310) = 6.85, *p* < .001, BF_10_ > 1000), and the real mask condition (*M* = 78.87%, *t*(310) = 8.55, *p* < .001, BF_10_ > 1000). The comparison between superimposed and real masks was also significant *t*(310) = 2.46, *p* = .042, BF_10_ = 1.25. There was a significant main effect of participant group whereby the super-recognisers as a group performed more accurately (*M* = 87.35%) than the control participants (*M* = 78.70%, *F*(3, 309) = 51.55 *p* < .001, *η_p_*^2^ = .14, BF_10_ > 1000. The interaction was non-significant *F*(3, 618) = 0.94, *p* = .392, *η_p_*^2^ < .01, BF_10_ = 0.23).

***Mismatch trial accuracy***

A mixed ANOVA with the within subjects factor of mask condition (unconcealed, superimposed mask, real mask) and the between subjects factor of participant group (control, super-recogniser) revealed a significant effect of mask condition on mismatch trial accuracy, *F*(3, 618) = 26.90, *p* < .001, *η_p_*^2^ = .08, BF_10_ > 1000, see Supplementary Figure 2c. Bonferroni corrected post-hoc comparisons showed that mismatch accuracy was significantly higher in the unconcealed condition (*M* = 89.68%) compared to both the superimposed mask condition (*M* = 84.95%, *t*(310) = 5.34, *p* < .001, BF_10_ > 1000), and the real mask condition (*M* = 82.89%, *t*(310) = 7.05, *p* < .001, BF_10_ > 1000). The comparison between superimposed and real masks did not survive correction *t*(310) = 2.16, *p* = .096, BF_10_ = 0.62. There was a significant main effect of participant group whereby the super-recognisers as a group performed more accurately (*M* = 92.11%) than the control participants (*M* = 80.97%, *F*(3, 309) = 87.06 *p* < .001, *η_p_*^2^ = .22, BF_10_ > 1000. The interaction was non-significant *F*(3, 618) = 1.27, *p* = .281, *η_p_*^2^ < .01, BF_10_ = 0.42).
